# Developing benzylisoquinoline alkaloid-enriched opium poppy via CRISPR-directed genome editing: A review

**DOI:** 10.1186/s12870-024-05412-x

**Published:** 2024-07-24

**Authors:** Zahra Aghaali, Mohammad Reza Naghavi

**Affiliations:** 1 Department of Genetics and Plant Breeding, Faculty of Agriculture, Tarbiat Modares University, Tehran, Iran; 2 Division of Plant Biotechnology, Department of Agronomy and Plant Breeding, College of Agricultural and Natural Resources, University of Tehran, Karaj, Iran

**Keywords:** Benzylisoquinoline alkaloids, CRISPR/Cas9, Genome editing, miRNA, Opium poppy, SNPs

## Abstract

Among plant-derived secondary metabolites are benzylisoquinoline alkaloids (BIAs) that play a vital role in medicine. The most conspicuous BIAs frequently found in opium poppy are morphine, codeine, thebaine, papaverine, sanguinarine, and noscapine. BIAs have provided abundant clinically useful drugs used in the treatment of various diseases and ailments With an increasing demand for these herbal remedies, genetic improvement of poppy plants appears to be essential to live up to the expectations of the pharmaceutical industry. With the advent of clustered regularly interspaced short palindromic repeats (CRISPR)/CRISPR-associated9 (Cas9), the field of metabolic engineering has undergone a paradigm shift in its approach due to its appealing attributes, such as the transgene-free editing capability, precision, selectivity, robustness, and versatility. The potentiality of the CRISPR system for manipulating metabolic pathways in opium poppy was demonstrated, but further investigations regarding the use of CRISPR in BIA pathway engineering should be undertaken to develop opium poppy into a bioreactor synthesizing BIAs at the industrial-scale levels. In this regard, the recruitment of RNA-guided genome editing for knocking out miRNAs, flower responsible genes, genes involved in competitive pathways, and base editing are described. The approaches presented here have never been suggested or applied in opium poppy so far.

## Introduction

Benzylisoquinoline alkaloids (BIAs) are among the most structurally diverse group of plant secondary metabolites containing nitrogen in their scaffold, with nearing the 2,500 known structures [1]. As secondary metabolites, BIAs make a significant contribution to the plant's chemical defense against abiotic and biotic stresses, including water scarcity, salinity, herbivores, and pathogens [2]. BIAs are also believed to have repellent properties against pests [3, 4]. Likewise, the propagation of microorganisms and viruses is hindered by BIAs [5]. Interestingly, BIAs function as the major players in plant acclimatization to the ever-changing environment [6]. Among characterized BIAs, morphine, codeine, thebaine, papaverine, sanguinarine, berberine, and noscapine have attracted much more attention since they bring considerable medical benefits to humans (Table 1).

**Table 1:** Pharmaceutical uses of benzylisoquinoline alkaloids massively produced in the different opium poppy tissues

Alkaloid	Type	Formula	Tissue	Property	Reference
Morphine	Morphinan	C_17_H_19_NO_3_	Ripe capsule	Analgestic Sedative Antidiarrheal	[7]
Codeine	Morphinan	C_18_H_21_NO_3_	Ripe capsule	Muscular relaxant Sedative	[8]
Thebaine	Morphinan	C_19_H_21_NO_3_	Ripe capsule	Analgesic	[9]
Papaverine	Papaverine	C_20_H_21_NO_4_	Unripe capsule	Antispasmodic Muscular relaxant	[10]
Sanguinarine	Benzo[*c*]phenanthridine	C_20_H_14_NO_4_	Root	Anti-inflammation Anti-cancer Anti-angiogenic Antiplatelet	[11–14]
Noscapine	Phthalideisoquinoline	C_22_H_23_NO_7_	Stem	Antitussive Anti-tumorigenic Anti-inflammatory	[15]

Ironically, little is known about their ecophysiological functions. Some studies document that the aforementioned BIAs play a crucial role in scavenging excessive free radicals generated as a result of various biotic and abiotic stresses [16, 17]. Sanguinarine and berberine put inhibitory effects on herbivores, bacteria, and fungi [5]. Berberine is recognized as a robust antifeedant compound preventing larval growth and feeding and subsequently reducing the population of pests such as fruit flies (*Drosophila melongaster*) [3, 4]. As a result of this, berberine has been applied to infected farmlands as a commercial insecticide [4]. While opium poppy plants are experiencing mechanical damage, morphine is being metabolized to bismorphine, which is integrated to the cell wall and confers resistance to hydrolysis mediated by pectinase [18].

BIAs are predominantly occurred in the Papaveraceae, Ranunculaceae, Berberidaceae, and Menispermaceae families [19]. Opium poppy (*Papaver somniferum* L.) belonging to the Papaveraceae family is assigned the commercial production of codeine, morphine, and the precursor thebaine. In addition, opium poppy is considered as the key supply of sanguinarine, noscapine, and papaverine. Thus, the pharmaceutical industries exclusively rely on opium poppy to satisfy their medical necessity. The chemical synthesis of most BIAs is far from being a possible option as they possess complex structures.

Opium poppy has been subjected to biotechnology-based approaches to boost the levels of pharmaceutically valued BIAs. This encompasses exposure to various biotic and abiotic elicitors and overexpression of BIA biosynthetic genes [20–25]. Likewise, the engineering of a rate-limiting enzyme, CYP82Y1, the construction of an artificial metabolon, and the overexpression of a WRKY transcription factor, *Ps*WRKY, are proposed as promising methods for BIA enhancement [26, 27].

Metabolic engineering had suffered from the scarcity of a technique enabling efficient and precise modulation of metabolite biosynthetic pathways until the advent of clustered regularly interspaced short palindromic repeats (CRISPR)/ CRISPRassociated9 (Cas9) for genome editing in 2012 [28]. Genomic mutations made by CRISPR are permanent and transferable to the posterities, providing a great opportunity for plant breeding [29]. More importantly, CRISPR-edited plants with optimized secondary metabolite profiles can be generated without becoming transgenic [30]. The application of CRISPR exceeds beyond gene knockout. It has also been utilized for single base-pair modifications, gene substitution, insertion and/or deletion, and gene expression enhancement [31]. CRISPR/Cas9 heads its site‐directed mutagenesis with the help of two components, an endonuclease and an RNA guide (gRNA) [28]. The most widely adopted endonuclease enzyme for the CRISPR-mediated editing is type II Cas9 derived from *Streptococcus pyogenes* (*Sp*Cas9). gRNA is responsible for identifying a short stretch of nucleotide bases, 3 bp, known as protospacer adjacent motif (PAM) and then binding to target DNA sequences which is adjacent to the PAM [32]. The resulting RNA–DNA complex becomes prone to the cleavage by Cas9 that introduces a double-strand break (DSB) within the genome. Subsequently, nonhomologous end-joining (NHEJ) machinery assumes the responsibility of repair [33], resulting in either completely accurate repair or insertion/deletion (InDel) mutations at the cleavage site [34].

Unfortunately, despite the importance of opium poppy as the sole source of industrial production of BIAs and its high status in today′s society as a model system for studying the biosynthesis of secondary metabolites, especially alkaloids, the applicability of the CRISPR/Cas9 system to promote BIA biosynthesis in this species has not yet been widely explored. The only effort in this regard is the knockout of *3′-hydroxy-N-methylcoclaurine 4′-O-methyltransferase* (*4′OMT*) producing a central branch point intermediate in BIA pathway, (*S*)-reticuline. In this study, sgRNA was transcribed from both viral-based (TRV) and synthetic binary plasmids and subsequently delivered into plant cells by *Agrobacterium* harboring a Cas9 encoding-synthetic vector. A dramatic decline in the levels of (*S*)-reticuline and BIAs was observed, confirming the regulatory role of *4′OMT* in BIA pathway [35].

The narcotic analgesics morphine and codeine are introduced as World Health Organization (WHO)-approved medicines owning to their worldwide utility as robust pain-relief drugs [36]. Despite this, more than 50 percent of individuals living around the world are deprived of effective remedies to treat moderate or severe pain [37]. In addition to producing important pharmaceuticals, opium poppy is a source of illicit drugs like heroin synthesized from morphine. Because of this, opium poppy is regarded as a double-edged sword, offering both beneficial and harmful compounds to human health [38]. Safer alternative platforms to producing opium poppy-derived medicinal agents are preferred but the occurrence of one or more chiral centers in the chemical structures of BIAs of medicinal significance hinders their large-scale chemical synthesis [39]. Until recently, extensive attempts have been made to synthesize morphine and its derivatives enzymatically, but none is currently applicable for the commercial production of them [40]. Regarding the microbial synthesis of opioids, both bacteria and fungi such as *Escherichia coli* and *Saccharomyces cerevisiae* have been engineered to assemble complex biosynthetic pathways of noscapine, sanguinarine, morphine, codeine, thebaine, and several derivatives. Nevertheless, although achieving significant progresses, synthetic biology is not yet a viable option for BIA industrial synthesis [41]. Taken together, opium poppy crops remain the sole commercial source of BIAs.

In this review, we discuss CRISPR/Cas9-associated strategies that target different genes involved in competitive pathways, adjusting flowering time, and miRNA biosynthesis. In addition, CRISPR-mediated modulation of SNPs named base editing is summarized. Noteworthy, the concurrent overproduction of all BIAs can be accomplished using the adoption of these strategies, avoiding labor-intensive and time-consuming step of increasing each BIA separately. Presumably, these proposed CRISPR-based approaches enable the development of methods for precision breeding of opium poppy tailored to the heightened BIA synthesis.

### Biosynthesis of major BIAs in opium poppy

**Fig. 1:**
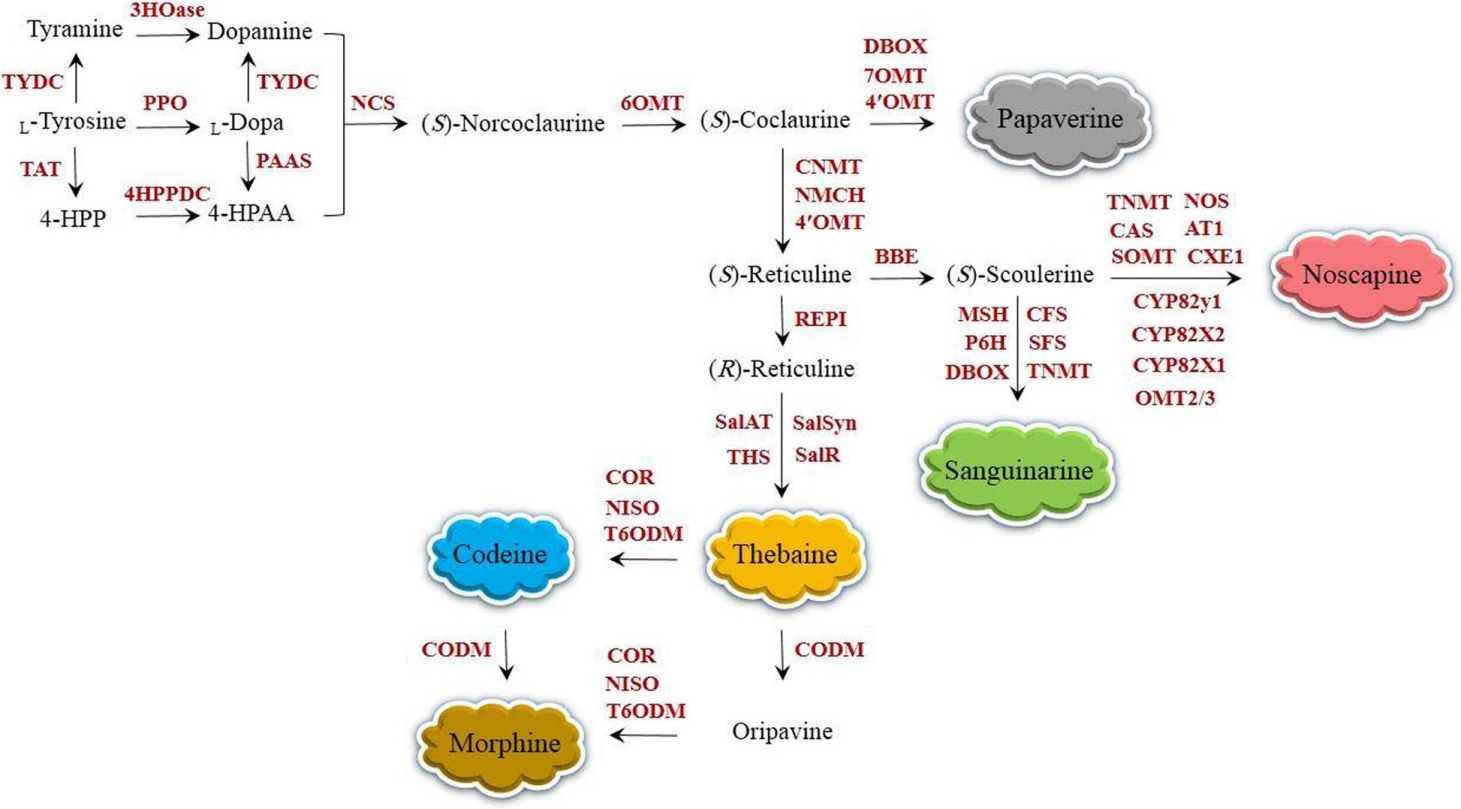
The biosynthetic pathways of dominant BIAs in opium poppy, leading to the formation of papaverine, noscapine, sanguinarine, thebaine, codeine, and morphine. All enzymes have been purified from opium poppy and functionally characterized. AT1, 1,13-dihydroxy-*N*-methylcanadine 13-*O*-acetyltransferase; BBE, berberine bridge enzyme; CAS, canadine synthase; CEX1, 3-*O*-acetylpapaveroxine carboxylesterase; CFS, cheilanthifoline synthase; CNMT, coclaurine *N*-methyltransferase; CODM, codeine *O*-demethylase; COR, codeinone reductase; CYP82X1, 1-hydroxy-13-*O*-acetyl-*N*-methylcanadine 8-hydroxylase; CYP82X2, 1-hydroxy-*N*-methylcanadine 13-*O*-hydroxylase; CYP82Y1, *N*-methylcanadine 1-hydroxylase; DBOX, dihydrobenzophenanthridine oxidase; 3HOase, _L_-tyrosine/tyramine 3-hydroxylase; 4-HPAA, 4-hydroxyphenylacetaldehyde; 4-HPP, 4-hydroxyphenylpyruvate; 4HPPDC, 4-hydroxyphenylpuruvate decarboxylase; MSH, *N*-methylstylopine 14-hydroxylase; NCS, norcoclaurine synthase; NISO, neopinone isomerase; NMCH, *N*-methylcoclaurine 3′-hydroxylase; NOS, noscapine synthase; OMT2/3, 4′-*O*-desmethyl-3-*O*-acetylpapaveroxine 4′-*O*-methyltransferase; 4′OMT, 3′-hydroxy-*N*-methylcoclaurine 4′-*O*-methyltransferase; 6OMT, norcoclaurine 6-*O*-methyltransferase; 7OMT, norreticuline 7-*O*-methyltransferase; PAAS, phenylacetaldehyde synthase; P6H, protopine 6-hydroxylase; PPO, polyphenol oxidase; REPI, reticuline epimerase; RNMT, reticuline *N*-methyltransferase; SalAT, salutaridinol 7-*O*-acetyltransferase; SalR, salutaridine reductase; SalSyn, salutaridine synthase; SanR, sanguinarine reductase; SOMT, scoulerine 9-*O*-methyltransferase; SPS, stylopine synthase; STOX, tetrahydroprotoberberine oxidase; T6ODM, thebaine 6-*O*-demethylase; THS, thebaine synthase; TNMT, tetrahydroprotoberberine *N*-methyltransferase; TYDC, _L_-tyrosine/DOPA decarboxylase; TAT, _L_-tyrosine aminotransferase

The biosynthesis of BIAs can be considered as an indication of exploiting resources efficiently. Despite considerable structural diversity, BIAs are biosynthetically originated from a single amino acid: _L_-tyrosine (Fig. 1). _L_-tyrosine undergoes the decarboxylation, *meta*-hydroxylation, and transamination, resulting in the formation of two intermediates dopamine and 4-hydroxyphenylacetaldehyde (4-HPAA) condensed by the first committed enzyme *(S)*-norcoclaurine synthase (NCS) (Fig. 1) [42]. The resulting *(S)*-norcoclaurine is converted to *(S)*-coclaurine by the enzyme norcoclaurine 6-*O*-methyltransferase (6OMT). The formation of* (S)*-coclaurine is the gateway reaction to papaverine synthesis (Fig. 1). Alternatively, *(S)*-coclaurine is transformed to *(S)*-reticuline through 3′-hydroxylation and consecutive *O*- and *N*-methylation reactions [39]. Different pathways, including morphinan (morphine, codeine, and thebaine), benzo[c]phenanthridine (sanguinarine), and phthalideisoquinoline (noscapine) are arisen from *(S)*-reticuline (Fig. 1) [39].

### CRISPR/Cas9-based strategies to enhance BIAs 

#### Repressing competitive pathways

**Fig. 2:**
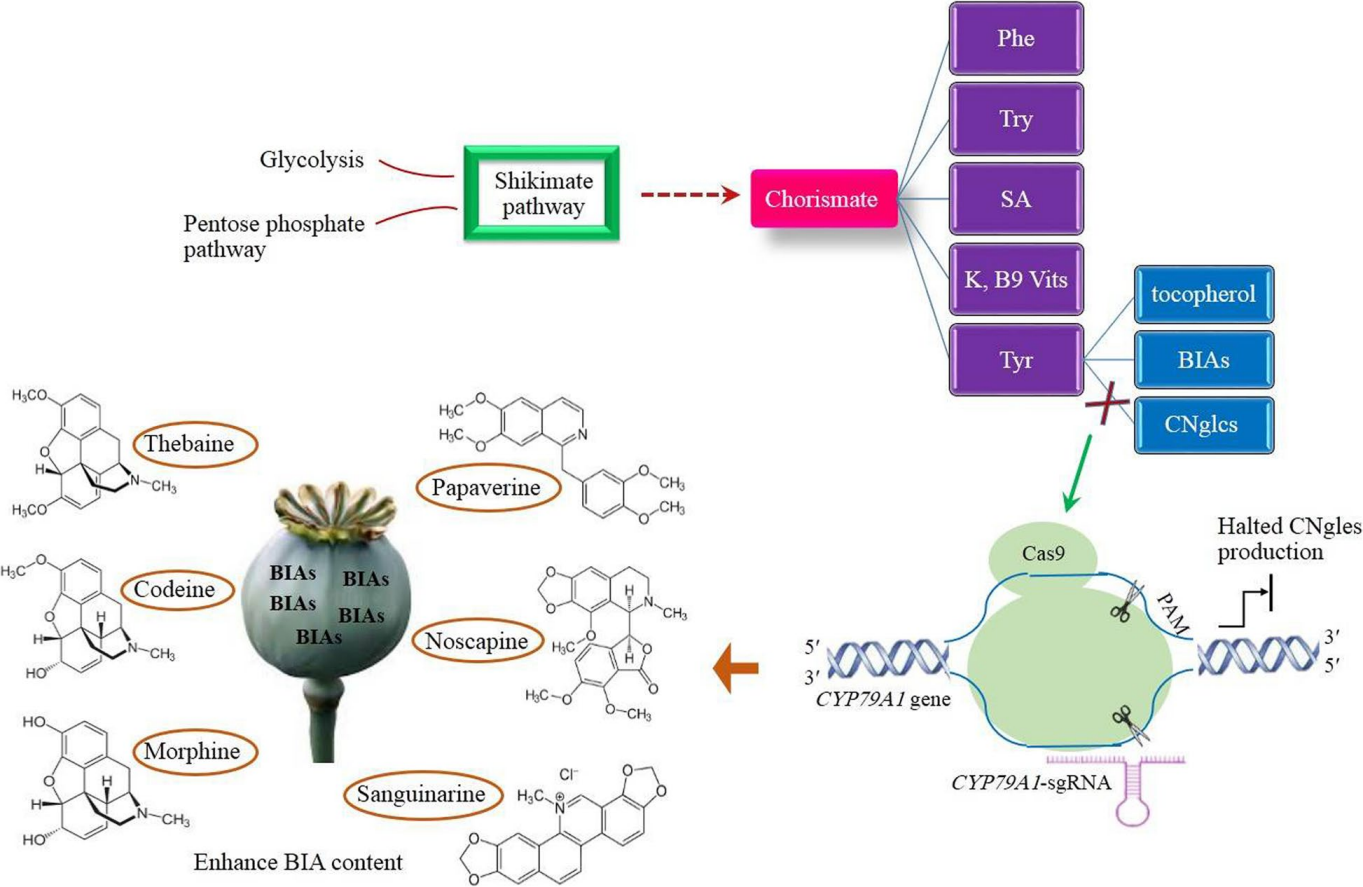
The shikimate pathway supports the synthesis of numerous primary metabolites. The final product of this pathway, chorismate, is channeled into the pathways generating K and B9 vitamins (Vits), _L_-tyrosine (Tyr), phenylalanine (Phe), salicylic acid (SA), and tryptophan (Trp). _L_-tyrosine is further converted to benzylisoquinoline alkaloids (BIAs), cyanogenic glycosides (CNgles), and tocopherol. The cleavage of *CYP79A1* involved in the biosynthesis pathway of CNgles by the means of the molecular scissor, Cas9, results in InDels in the given gene, generating loss-of-function allele. As a consequence, more _L_-tyrosine is directed toward BIA pathway

_L_-tyrosine, in addition to tryptophan and phenylalanine, results from chorismate which is in turn derived from the glycolysis and pentose phosphate pathways through the seven enzymatic reactions of shikimate pathway (Fig. 2) [43]. Chorismate has been recognized as the central intermediate in which amino acid biosynthetic pathways are diverged from it (Fig. 2). Chorismate serves as the origin of numerous primary metabolites, including vitamins K and B9 and the major defense-responsive hormone, salicylic acid, as well (Fig. 2) [44]. It has been documented that in the majority of circumstances, the central carbon flux acts in favor of phenylalanine biosynthesis. It means that the highest amount of chorismate is directed to its biosynthetic pathway to synthesize myriad phenylalanine–derived compounds, such as phenolics, lignin, flavonoids, and anthocyanin [43]. As a result, a much smaller flux flows towards _L_-tyrosine biosynthesis compared to that of phenylalanine.

In addition to BIAs, _L_-tyrosine has also been found to be consumed for the synthesis of tocopherol and cyanogenic glycosides (dhurrin) [43]. Tocopherol in plants is similar to vitamin E in animals in terms of biological activity. Both represent the antioxidant activity. The advantages of tocopherol offered to plants have been enormously studied. Plants containing sufficient levels of tocopherol strongly resist several abiotic stresses, such as water deficiency, salinity, UV radiation, extreme temperature, and so on [45]. Moreover, a pivotal role is played by tocopherol in physiological processes, including growth and development, signal transduction, phytohormonal regulation, and senescence [46].

Cyanogenic glycosides, also known as dhurrin, are biologically active compounds initially identified in *Sorghum bicolor*, but their occurrence has then been evidenced in excess of 3,000 plant species belonging to 130 families alike [47]. Cyanogenic glycosides actively participate in several biological processes, including seed germination, bud burst, and carbon and nitrogen transport across the cells [48]. Also, they participate in the mitigation of oxidative stress [49]. To generate dhurrin, the activity of CYP79A1 allows the entry of _L_-tyrosine to its biosynthetic pathway [50]. This rate limiting enzyme catalyzes the conversion of _L_-tyrosine to *p*-hydroxyphenylacetaldoxime [51]. Next, the resulting compound is transformed to dhurrin by CYP71E1 and UDP-glucosyltransferase (UGT85B1) [52].

It is ironic that despite the principle functions, cyanogenic glycosides are not counted as essential components in most environmental conditions [47]. Therefore, plant species can bear the elimination of cyanogenic glycosides and stay alive. However, the opposite has been reported for tocopherol. In other words, plants are highly susceptible to the reduction of tocopherol level and a reduction in its content poses adverse and irreversible effects on plant survivability and resilience [45]. In an experiment conducted to develop transgenic sorghum plants harboring reduced levels of cyanogenic glycosides, the expression of *CYP79A1* was down-regulated by antisense mediated. The dhurrin content in the transgenics reduced remarkably to 5.1 and 149 μg/g on dry weight basis in comparison with 192.08 μg/g in the non-transformed control. Moreover, progenies in T3 generation produced considerably reduced levels of dhurrin, 62.9 and 76.2 μg/g compared to 221.4 μg/g in the control plants [53].

Hence, to channel more _L_-tyrosine into BIA pathway, cyanogenic glycoside pathway, not tocopherol, should be repressed by CRISPR. Through knocking out *CYP79A1*, first-committed enzyme in cyanogenic glycoside biosynthetic pathway, no competition for _L_-tyrosine consumption occurs between cyanogenic glycoside pathway and the other two pathways, tocopherol and BIA, so that _L_-tyrosine is exclusively redirected toward BIA and tocopherol pathways. As a result, the pathway of BIA synthesis receives more _L_-tyrosine compared to before. The BIA content is expected to elevate as a consequence (Fig. 2).

### Delaying flowering time

Among floral attributes, flowering time stands out as one of the major determinants of the commercial-scale production of cut flowers, bioactive compounds, and even fodder. Indeed, flowering time ensures commercial success and economic gains. The transition from vegetative to productive stage occurs as a result of endogenous and exogenous cues. Several developmental and environmental signals impact the floral meristem [54]. In poppy plants, they mostly include, but are not limited to, temperature and photoperiod [55]. The flowering induction has been proven to be regulated by eight pathways, photoperiodic, autonomous, vernalization, hormonal, sugar, aging, and temperature. Although acting primarily dependently, these pathways interact with each other at times [54]. The most paramount regulator among genes involved in the floral transition pathways is *FLOWERING LOCUS T* (*FT*) [56]. Once translated in the leaf phloem, FT makes a journey to primary meristems where stimulates the expression of the other flowering induction genes, such as *SUPPRESSOR OF OVEREXPRESSION OF CONSTANCE 1* (*SOC1*) and *APETALA 1* (*AP1*) and the eight pathways mentioned previously, collectively inducing flowering [56].

Morphine, codeine, thebaine, sanguinarine, papaverine, and noscapine are abundantly occurring in vegetative, not productive, tissues, including mature capsule, root, unripe capsule, and stem. Flowers and seeds of poppy are deprived of BIAs [57]. Thus, breeding for the prolonged vegetative growth will contribute to the robust vegetative organs, extended harvest time, improved quality, higher biomass yield, and more importantly, higher BIA biosynthesis and accumulation. Functional information about gene networks associated with flowering enables manipulation of key genes in this process. Owning to the leading function of *FT* in flowering, its genetic alteration should be on the priority over other genes in this process. The high percentage of similarity with other proteins necessitates the sequence-specific and targeted editing of *FT* gene [58]. With the advent of CRISPR/Cas9 methodology, this goal is achievable via designing sgRNA unique to the *FT* sequence. *FT* mutagenesis could result in the hindrance of the expression of downstream genes acting in pathways that directly or indirectly affect flowering, preventing the transfer of the floral signal to primary meristems. Consequently, an extension of the vegetative growth and an enhancement in BIA quantity may be witnessed.

### Suppressing microRNAs

MicroRNAs (miRNAs) are denoted as indigenous small, double-strand, non-protein-coding RNAs that are transcribed from *MIR* genes, with the maximum length of 23 nucleotides. Almost all processes that happen in a living organism bear a trace of miRNAs. miRNAs post-transcriptionally regulate the expression of genes involved in multitude developmental and biological activities, such as seed emergence, blooming, cell and tissue differentiation, apoptosis, signal transductions, phytohormone pathways, primary and secondary metabolisms, and responses to biotic and abiotic stresses in opium poppy [59, 60]. They bind with their complement binding sites situated within target mRNAs, resulting in the degradation or translation inhibition of the transcript via the RNA interference mechanism [61]. Interestingly, it has been recently found that miRNAs can assume the function of signaling molecules. In this situation, the communication between plants and interacting organisms can be established by exchanging miRNAs, allowing plant-to-plant and host-to-microbe interactions [62]. miRNAs and their binding sites act in a species-specific manner. In other words, they are conserved in a given species [63]. In spite of identification of numerous miRNAs in plants, there is insufficient evidence regarding miRNAs wrecking the control of secondary metabolite biosynthesis [64, 65]. Among up to 327 tissue-specific miRNAs in poppy plants, those that influence BIA biosynthesis have recently been functionally characterized (Table 2) [66]. The pso-miR2161 and pso-miR13 prevent the transcripts of 4′OMT and 7-*O*-methyltransferase (7OMT) from being translated, respectively (Table 2) [66]. The former enzyme catalyzes the methylation of 3′-hydroxy-*N*-methylcoclaurine, but the latter performs the same reaction on (*S*)-reticuline [67, 68]. mRNAs encoding codeinone reductase (COR) and salutaridinol 7-*O*-acetyltransferase (SalAT) are evaluated to be on the tight control of pso-miR t0047847 and pso-miR t0013376, respectively, whereas the _L_-tyrosine/_L_-DOPA decarboxylase (TYDC) transcript is inhibited by pso-miR t0000199 (Table 2) [66]. COR and SalAT undertake the responsibility of converting (*S*)-reticuline to morphinan alkaloids [69, 70], and the decarboxylation of both _L_-tyrosine and _L_-DOPA is mediated by TYDC [71]. Correspondingly, pso-miR408 is predicted to target the mRNAs of FAD-binding and berberine bridge enzyme (BBE) domain-containing protein (Table 2) [66], which generate (*S*)-scoulerine from (*S*)-reticuline [72].

**Table 2:** Conserved miRNAs targeting varied BIA-biosynthesis genes and pathways

Name	Target transcript	Target pathway
pso-miR2161	4′OMT	All BIAs
pso-miR13	7OMT	All BIAs except papaverine
pso-miR t0047847	COR	Morphinan
pso-miR t0013376	SalAT	Morphinan
pso-miR t0000199	TYDC	All BIAs
pso-miR408	FAD-binding and BBE	Noscapine and sanguinarine

7OMT 7-*O*-methyltransferase, *BBE* berberine bridge enzyme, *COR* codeinone reductase, *SalAT* salutaridinol 7-*O*-acetyltransferase, *TYDC*_L_-tyrosine/_L_-DOPA decarboxylase

Till date, CRISPR has been extensively adopted for the knockout of biosynthetic gene. However, a novel avenue has been opened to researchers to explore whether *MIR* genes can be subjected by this technique. The employment of CRISPR/Cas in miRNA coding genes to regulate metabolite biosynthetic pathway is promising and feasible; however, the modification of target MIRs is in its infancy and still challenging. miRNAs are resistant to frameshift mutations induced by one sgRNA [73]. For this reason, the application of dual sgRNAs to simultaneously edit both ends of a miRNA gene would be effective [74]. This results in the elimination of whole *MIR* gene or the impairment of the processing of miRNA [74]. The reliance on the 5′-NGG PAM sequence required for the classic Cas9 is restricted the usage of the CRISPR/Cas9 system to fewer *MIR* genes since due to the small size of miRNA genes, the 5′-NGG PAM site may not be found in all miRNA genes [75]. However, various Cas protein variants have been developed to address this issue. For instance, Cas9 nucleases from *Staphylococcus aureus* (*Sa*Cas9), *Neisseria meningitides* (*Nm*Cas9), and *Campylobacter jejuni* (*Cj*Cas9) can recognize NNGRRT, NNNNGATT, and NNNNACAC PAMs, respectively [75, 76]. Recently, a near-PAMless Cas9 variant termed *Sp*RY is engineered to target NRN and NYN sequences [77]. Therefore, *MIR* genes can be modified as effective as protein-coding genes through employing these optimized Cas variants.

Single bp InDels introduced by CRISPR/Cas9 often reduce disruption to miRNA activity [78]. The solutions to this issue are to generate medium or large fragment deletions through inducing two DSBs and to increase the frequency of deletions at target miRNA gene [78, 79].

Until recently, the CRISPR/Cas-based miRNA editing has only been utilized in plant species *Arabidopsis thaliana* [80], *Marchantia polymorpha* [81], rice [80], wheat [82], soybean [83], and tomato [84]. The efforts to knockout *MIR* genes has been restricted to agronomically important trains, including higher yield, nutrition acquisition efficiency, and enhanced resistance against biotic and abiotic stresses [85]. Therefore, further studies on the identification and functional analysis of miRNAs involved in biosynthetic pathways of secondary metabolites as well as on the application of CRISPR-based technologies are necessary to routinely employ this technology in metabolic engineering in medicinal plants, especially opium poppy.

Moreover, given that miRNAs act cooperatively to modulate different pathways and in some cases, a single miRNA affects the expression of another miRNA [86], it is essential to determine whether the identified miRNAs in opium poppy work independently to precisely manipulate these miRNAs through genome editing. Another issue is that *MIR* genes are frequently located in fragile regions like exons or regulatory regions of protein-coding genes so their editing needs special care [86]. Hence, prior to editing of *MIR* genes in opium poppy, the distribution pattern of them in the genome is required to avoid destructing genes encoding proteins.

**Fig. 3:**
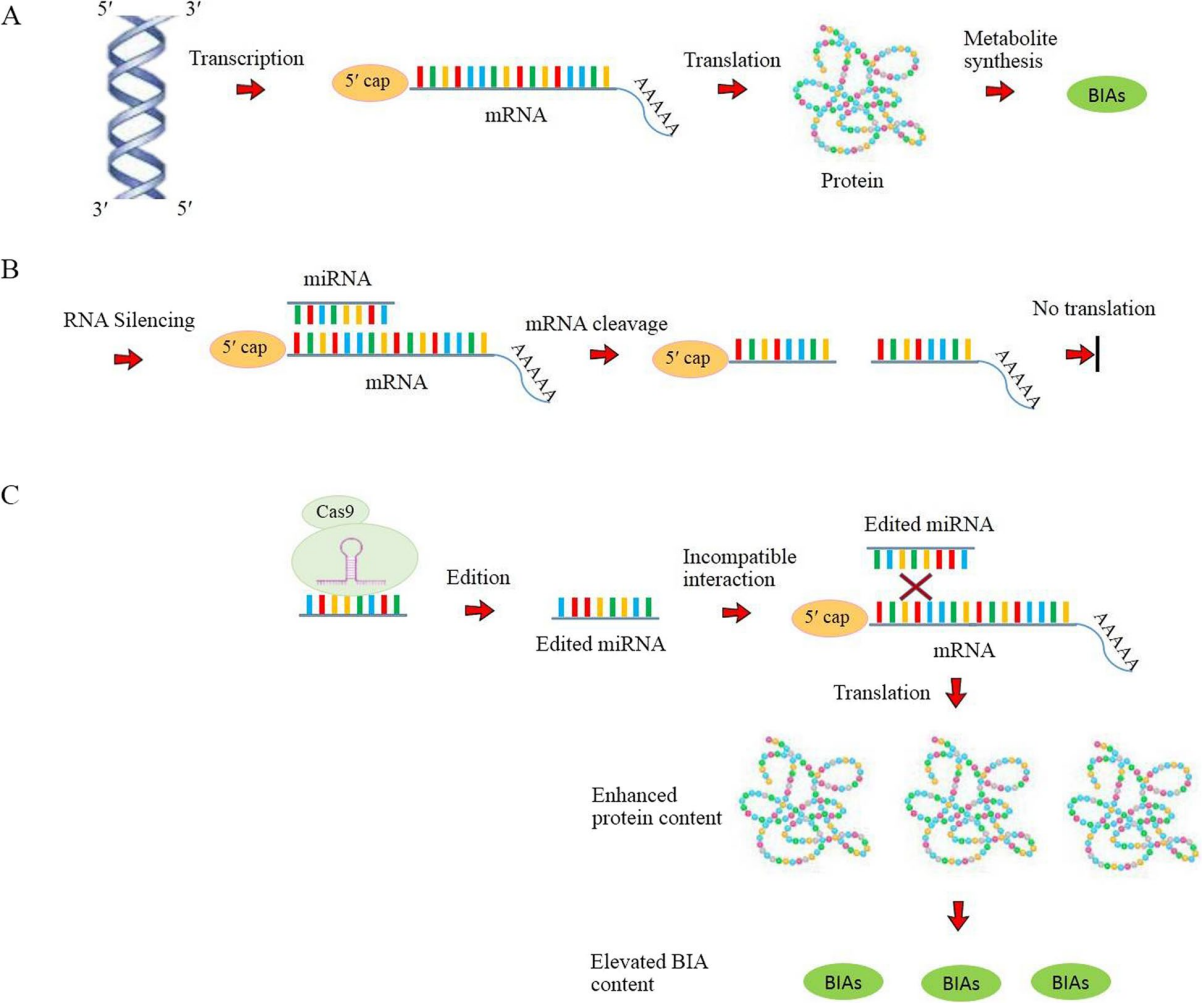
**a** genetic information archived in DNA is transferred to mRNA transcript and to protein that finally gives instruction to secondary metabolism responsible for BIA biosynthesis. **b** mature mRNA processed by 5′ capping and 3′ polyadenylation is directed to the RNA-silencing pathway mediated by miRNAs. The miRNA complementary to mRNA inhibits its translation via cleavage the transcript. **c** CRISPR-based miRNA editing causes the modifications in the mRNA sequence. The resulting miRNA is incapable to bind with the target mRNA, culminating in the continuous protein synthesis and an improvement in the BIA accumulation.  Adenine;  Uracil;  Cytosine;  Guanine

A schematic overview of the mechanism in which miRNA editing promotes the production of end products is illustrated in Fig. 3. When *MIR* genes are exposed to genetic mutations introduced by CRISPR/Cas, target gene′s transcripts are released from the inhibition of this miRNA. As a result, the gene is expressed constitutively and the abundance of its transcripts are continuously increasing. The translation of these transcripts provides higher precursor for downstream enzymes, resulting in a substantial increase in the level of end product, e.g. BIAs (Fig. 3).

### Base editing

The assessment of plant metabolic diversity occupies an important position in plant breeding procedures. With the advent of metabolic profiling technologies, the interspecific and intraspecific variations of metabolites have been analyzed quantitatively [87]. Genome-wide association study (GWAS) accompanied by metabolomics broaden our knowledge regarding the genetic basis of plant metabolism via the determination of DNA variants having enormous importance on metabolic fluctuations [88]. GWAS explores a plethora of genetic loci associated with complex traits, such as metabolite of interest, facilitating the identification of the gene–metabolite association [88]. In poppy plants, just two molecular markers, including amplified fragment length polymorphism (AFLP) and simple sequence repeat (SSR), have been employed for the detection of the genomic regions linked to BIA content [89, 90]. Nevertheless, with the arrival of high-throughput sequencing technologies, differentiation of poppy chemotypes according to single nucleotide polymorphisms (SNPs) is on the rise. As the most pervasive markers in plant genomes, SNPs supply deeper and more comprehensive understanding of molecular mechanisms of plant metabolism. As well as that, SNPs enjoy high sensitivity, robustness, quality assurance, and commercial effectiveness [91]. The recognition of SNP variants responsible for differences in alkaloid content in poppy has been allowed by GWAS [92].

**Fig. 4:**
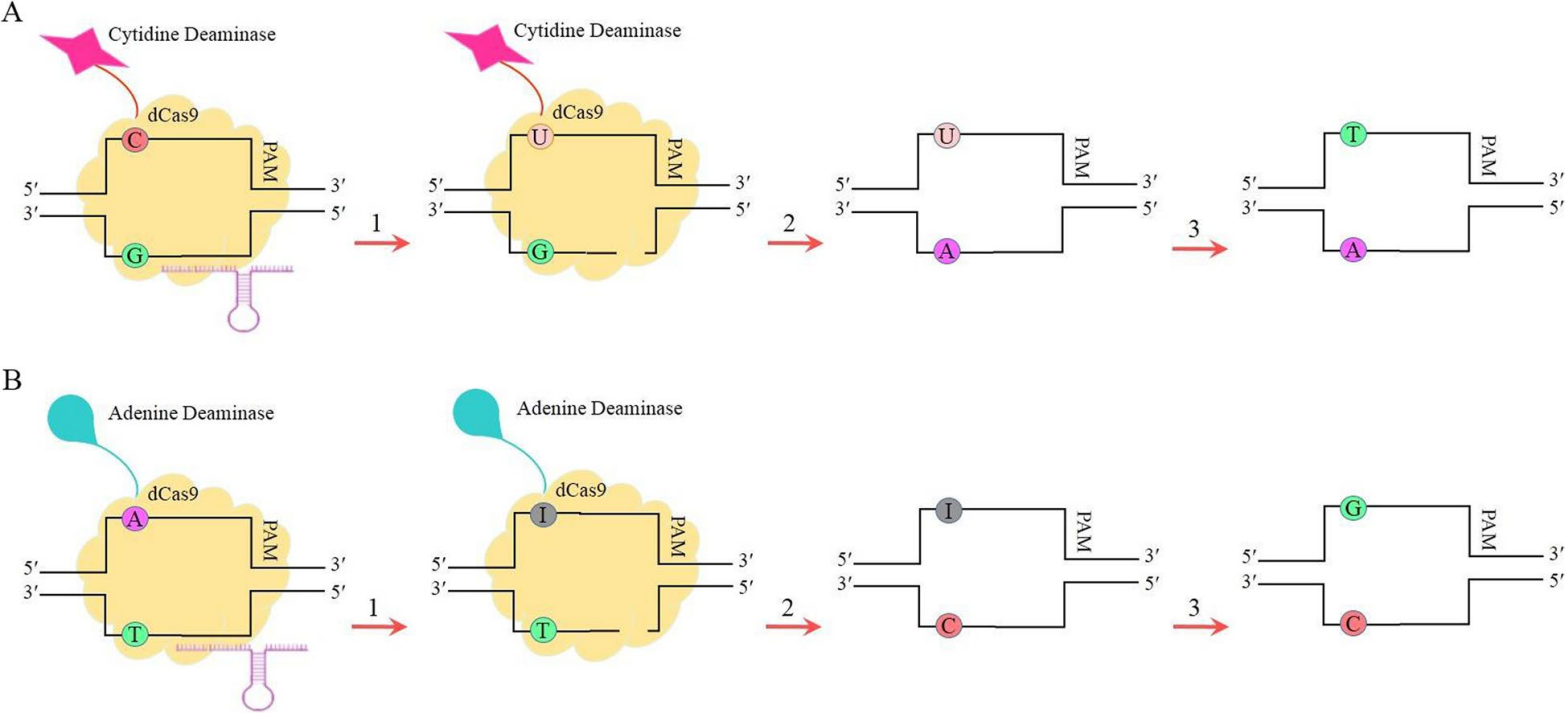
The schematic overview of base editing. **a** cytosine base editor (CBE) enables the conversion of C to uracil (U) directed by cytidine deaminase (1). The resulting unmatched U*G is transformed to U*A pair after DNA repair on the non-edited strand (2). Ultimately, T*A pair is resulted from DNA replication (3). **b** adenine base editor (ABE) makes use of adenosine deaminase instead. The enzyme converts A to inosine (I) (1). The I*T base mismatch is corrected to I*C pair by repair mechanism on the non-edited strand (2). The final G*C pair is produced during DNA replication (3)

Given the fact that SNPs are underlying contributors to the BIA variation, they can be considered as one of the best options for editing by CRISPR. The CRISPR/Cas9 system relies on homology-directed repair (HDR) to introduce a desired alteration to a single base pair [93]. Since a competition between HDR and NHEJ almost always takes place at the DSB site and usually terminates in favor of NHEJ [94], a number of nucleotides are excluded from the genome. Hence, owning to the high frequency of InDel mutations, the endonuclease-based genome editing is not applicable for editing single base pair. A revised version of CRISPR, therefore, which avoids generation of DSB turns out to be essential. Base editing stands out as one of the most viable approaches that can specifically modify the sequence of a single-nucleotide [95]. To do so, two base editors have been optimized recently: Cytosine base editor (CBE) and adenine base editor (ABE) are assigned to the conversion of C to T and A to G and vice versa, respectively [95]. CBE and ABE share a catalytically impaired Cas9 nuclease (dCas9) fused to a base modification enzyme (cytidine deaminase or adenine deaminase). CBE is additionally composed of a uracil glycosylase inhibitor [95]. The modified Cas9 nicks the DNA strand opposite the PAM-containing one, providing the opportunity for the deaminases that are naturally act on single-strand DNA to catalyze the deamination reaction [34]. The underlying mechanisms whereby these base editors amend a single-point mutation are detailed in Fig. 4. To the best of our knowledge, base editing has not yet been applied for enhancing the contents of secondary metabolites of medical values. However, in several crops, CBE and ABE are currently used to improve agronomic traits, such as herbicide resistance, nutrition acquisition, flowering time, and grain yield [96]. With precise converting C to T at position P197 of *Brassica napus acetolactate synthase* (*BnALS1*) gene using CBE, a novel herbicide resistance oilseed rape was generated [97]. In *Arabidopsis thaliana* and *Brassica napus*, A-to-G replacement in the FT protein using ABE resulted in plants with late flowering [98].

In BIA biosynthetic pathway, NCS has been found to catalyze the rate-limiting step [99]. Thus, the efficiency of (*S*)-norcoclaurine biosynthesis and the control of metabolic flux strongly rely on the catalytic efficiency of NCS [99]. In a study, through homology-based modeling and molecular dynamic, the structure of *Papaver somniferum* NCS (*Ps*NCS), the impact of the residues residing around the active site on the activation energy, and the rate-enhancing mutations were predicted [100]. The charged residues that negatively affect the efficiency of enzyme were identified as Asp64, Asp71, Arg105, Lys110, Glu117, and Lys163, suggesting the favorable candidates for mutation. Since the residues were suggested to be localized in the enzyme’s surface, their charged groups face the surrounding solvent and do not interact with other residues via hydrogen bridges. They, therefore, guarantee the solubility of NCS. This provides a unique opportunity for mutation. If they are substituted with polar or oppositely charged residues, the structure of NCS will not be disturbed [100].

The findings of this study laid the groundwork for base editing of NCS in further experiments. The enzyme may bear the substitutions of Glu to Gln, Asp to Asn, Arg to Lys, and Lys to Gln because they differ only in a single base pair, For example, Lys is encoded by AAA/AAG but GLn is resulted from CAA/CAG. Moreover, in these SNP mutations, the former residue and the replaced one are both polar so the polarity of the environment surrounding residues is retained [100]. Additionally, although the side chains charge is removed, other characteristics of the residues remain unchanged and the structure of NCS may not be disturbed [100]. Consequently, the catalytic activity of NCS will be enhanced without any structure rearrangement, resulting in higher BIA production.

Single amino acid substitution using base editing is favored to computational design methods due to its independency from computer simulations, bioinformatics methods, and prior knowledge regarding protein structure, the structure–function relationship, accurate protein modelling, and intermolecular interactions.

### Challenges in engineering BIA pathway by applying CRISPR/Cas technology

The availability of opium poppy reference genome sequence accounting for up to 95% of the whole genome size facilitates the applicability of CRISPR/Cas9-based genome editing in this plant.

However, enhancing BIA content with the help of CRISPR faces some hurdles that should be overcome. With regard to off-target effects, similarity in gene sequences limits its application [101]. Phylogenic analysis identified that morphinan pathway genes have a high degree of sequence homology, reflecting their evolutionary history. Interestingly, codeine 3-*O*-demethylase (CODM) and thebaine 6-*O*-demethylase (T6ODM) share more that 97% amino acid identity [102]. Thus, the target specificity of sgRNAs designed to edit genes involved in morphinan pathway must be strictly validated.

Based upon a logistic regression model, off-target occurrence is believed to be mainly impacted by five factors: mismatch frequency, mismatch position, GC-content, nuclease variants, and delivery methods [101]. The number mismatches is reversely correlated with off-target effects. Off-target genome editing is reduced to up to 59% when a single mismatch is present [103]. However, location of mismatches is more important than their quantity. Mismatches at the 5′ end of sgRNAs, even three to five base pair mismatches, can still contribute to off-target cleavage activity [104]. Due to close homology between *4*′*OMT* and* 4*′*OMT1*, *4*′*OMT*-targeting sgRNA showed 17 bp matches out of 20 bp with *4*′*OMT1* at the 5′ end. The presence of mismatches at the 3′ end was not tolerated by the CRISPR/Cas9 system and therefore, off-target cleavage was inhibited [35].

GC content is another principal factor influencing off-target gene editing. Increasing GC content positively affects on-target activity since higher GC content stabilizes the DNA: RNA hybridization [103]. Although GC contents of BIA biosynthetic genes are determined and are available in the web-based NCBI database, the impact of GC content on the gene editing efficacy in opium poppy needs to be unveiled. The current understanding of the endonuclease variants and delivery techniques on off-target effects is finite [105].

Establishing an efficient procedure for the transformation and regeneration is a significant obstacle to the application of CRISPR/Cas9 in opium poppy. Opium poppy has been recognized as a difficult to transform with *Agrobacterium* and very recalcitrant plant species because of the massive amount of alkaloids that hinder transformation and differentiation of plant cells [106]. Moreover, all regeneration protocols vary depending on varieties and genotypes [107]. To overcome these limitations, nanoparticle-based plasmid delivery and polyethylene glycol-mediated protoplast transformation have been proposed [108].

Other underlying contributors, including promoters, terminators, selectable markers, plasmid vectors, and driving machinery should also be taken into account to witness the effective utilization of the CRISPR tool in opium poppy.

### Ethical issues surrounding gene editing

In recent years, agriculture and the environment have experienced advances in CRISPR/Cas-mediated gene editing. However, this technology has raised some legal and ethical issues on these sectors. Organic farming communities argue that gene editing disturbs the natural evolutionary processes via the conversion of wild-type alleles into drive alleles in a wild-type population, changing heterozygous to homozygous [109]. In RNA-targeted gene editing, the occurrence of off-target mutations is undeniable. This may result in a set of concomitant disadvantages. This unintentional failure will persist in each generation [110]. The mutations may be transferred to future generations. The frequency of mutations may increase as time passes [111]. The alleles with non-target modifications may be disappeared absolutely due to gene drift. More importantly, horizontal gene transfer may occur between the plant carrying off-target mutations and other living beings in the environment, increasing the possibility of the transmission of negative attributes to the associated organisms [112].

Gene editing applications should be in the realm of biosafety law to ensure the safety of people and the environment. When not observing legal and ethical regulations, the agricultural gene editing practices are not allowed to leave the laboratory. Nevertheless, from the perspective of the proponents of gene editing, gene-edited products are not genetically transformed with foreign genes, transgenes, thus, biosafety regulations should not be implemented on them [113]. On the other hand, the critics questioned the biosafety of gene editing because of very limited understanding about off-target effects [114]. Fortunately, newly developed CRISPR-Cas is capable of minimizing or even preventing non-target gene editing.

Due to the controversies about CRISPR/Cas, regulatory policies are required to be developed for future processing, research and development, and commercial uses [115]. Notably, rules and regulations should be clear, science-based, effective, defendable, credible, and proportional to the context which is planned to use to be accepted by the scientific community and bioethical and legal parties [109].

### Concluding remarks and future perspectives

CRISPR-oriented metabolic engineering has emerged as a promising tool to tackle the insufficiency of opium poppy-derived medicines through introducing preferred and genome-wide genetic changes in the genome. However, future efforts must be focused on uncovering the untapped potentialities of CRISPR/Cas9. CRISPR/Cas9-based genome editing could counteract the negative characteristics of conventional methods for metabolic engineering suffering from accidental gene insertion, unpredicted results, position effect, and low efficiency. It is worth mentioning that genome editing by CRISPR/Cas9 is not limited to developing poppy plants with advanced pharmaceutical outputs; it can expand its applications to a range of traits beneficial to farmers and the food industry by creating biotic and abiotic stress-resilient genotypes, plants with delayed senescence, and essential oil-enriched seeds, respectively.

The global trend toward synthetic drugs has been reversed. The popularity and acceptability of herbal-based medicines such as opium poppy-extracted BIAs are continuously increasing since they offer a safe, inexpensive, easy to access, and no side-effects therapy [15]. Persons of all ages and genders have received herbal therapy at least once in their lives. A surprising number of people are treated with codeine or strong opioid like morphine, 69% and 99%, respectively [116]. The number of patients whose principal drugs of concern are BIAs is growing. Hence, it is apparent that the pharmaceutical companies will require a substantial amount of BIAs to meet the increasing demand of practitioners and patients as well. Additionally, with the continuous advent of novel strains of pathogenic microorganisms, the development of new drugs is urgent. Because the efficacy of BIAs against emerging diseases, such as SARS-COVID 9 and HIV has been proven [117–119], it can be projected that BIAs efficiently will combat unprecedented health-threatening problems in the upcoming years. Therefore, the exploitation of strategies enhancing their production is rational and necessary.

Furthermore, BIAs can supply raw materials for fabrication of semisynthetic derivatives. Thebaine, as an example, is used as the starting material for the semi-synthesis of oxycodone and hydrocodone mitigating acute pains; naltrexone reducing opioid cravings; and naloxone reversing opioid overdose [39].

The advancements in functional genomics tools, including VIGS-mediated analysis, transcriptomics, proteomics, and next-generation sequencing (NGS) projects have broaden our knowledge of the organization of BIA biosynthetic genes and enzymes, BIA metabolic networks, and regulatory mechanisms at different levels. These achievements are likely to substantially enhance the effectiveness of innovative metabolic engineering strategies like CRISPR/Cas and to provide an opportunity for BIA industrial biosynthesis in opium poppy.

## Data Availability

No datasets were generated or analysed during the current study.
